# Multiple myeloma concealed by adrenal Cushing syndrome: a case report and review of the literature

**DOI:** 10.1186/s13256-018-1731-y

**Published:** 2018-08-13

**Authors:** Taweesak Wannachalee, Nuttagarn Jantanapornchai, Kittima Suphadirekkul, Sirinart Sirinvaravong, Weerapat Owattanapanich

**Affiliations:** 1 Division of Endocrine and Metabolism, Department of Medicine, Faculty of Medicine, Siriraj Hospital Mahidol University, Bangkok, Thailand; 2 Sena Hospital, Phra Nakhon Si Ayutthaya, Thailand; 3 Division of Hematology, Department of Medicine, Faculty of Medicine Siriraj Hospital, Mahidol University, Bangkok, Thailand

**Keywords:** Multiple myeloma, Cushing syndrome, Adrenocortical adenoma, Adrenalectomy

## Abstract

**Background:**

Cushing syndrome coexisting with multiple myeloma has been previously described in a few reports. Overlapping clinical manifestations can lead to misdiagnoses.

**Case presentation:**

We presented an extremely rare case of a 33-year-old Thai woman with concomitant kappa light chain myeloma with adrenal Cushing syndrome, both of which were related to skeletal manifestations. A precedence report indicated that treatment of the Cushing syndrome could exacerbate the myeloma symptoms. Therefore, we were faced with the dilemma of which disease should be addressed first. We decided to treat our patient with a combination chemotherapy followed by an autologous stem cell transplant. Subsequently, a left laparoscopic adrenalectomy was successfully undertaken.

**Conclusion:**

We have reported the first association between adrenocorticotropic hormone-independent Cushing syndrome resulting from a left autonomous cortisol-secreting adrenal adenoma, and multiple myeloma.

**Electronic supplementary material:**

The online version of this article (https://doi.org/10.1186/s13256-018-1731-y) contains supplementary material, which is available to authorized users.

## Background

Cushing syndrome (CS) is a serious condition resulting from chronic exposure to cortisol excess, and it is associated with increased risks of cardiovascular, metabolic, and psychiatric complications, and osteoporosis. The causes of endogenous hypercortisolism are related to an overproduction of cortisol by the adrenal glands or of adrenocorticotropic hormone (ACTH) by the pituitary gland and ectopic sources [1, 2].

Turning to another disease, multiple myeloma (MM) is the second most common hematologic malignancy; it is caused by abnormal plasma cells, which produce monoclonal immunoglobulin. The clinical signs and symptoms include hypercalcemia, renal failure, anemia, bone pain, and pathological fractures. The standard treatment for MM consists of combination chemotherapies, which are usually combined with corticosteroid in most regimens [3].

The concomitant occurrence of endogenous CS (ECS) with MM is exceedingly rare. Some of the clinical manifestations of the two diseases overlap, which lead to misdiagnoses by clinicians. Moreover, the hypercortisolemic state could suppress the presentation of the MM.

## Case presentation

A 33-year-old Thai woman presented with progressive back and arm pain for 2 weeks. Her body weight (BW) had increased by 10 kg over the preceding 2-year period, and she had also noticed dark striae on her abdominal wall. She complained of excessive acne on her face, but she still had normal menstrual cycles and no hirsutism. She had not visited a hospital about the symptoms before. Then, 2 weeks prior to admission, she had a non-severe falling accident, but she still had worsening back and right arm pain. She also had a history of occasional use of Chinese herbs and weight loss pills. On examination, a rounded face, truncal obesity, and wide purplish striae on her abdominal wall and right thigh were observed. Her blood pressure was 160/90 mmHg. CS was therefore suspected.

**Fig. 1:**
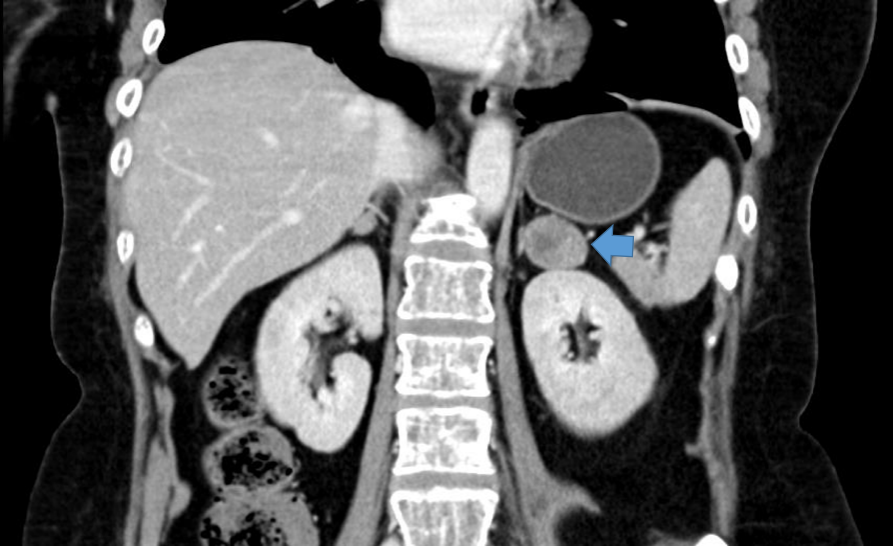
Computed tomography showing a lipid-poor left adrenal adenoma of size 2.8 cm (arrow)

**Fig. 2:**
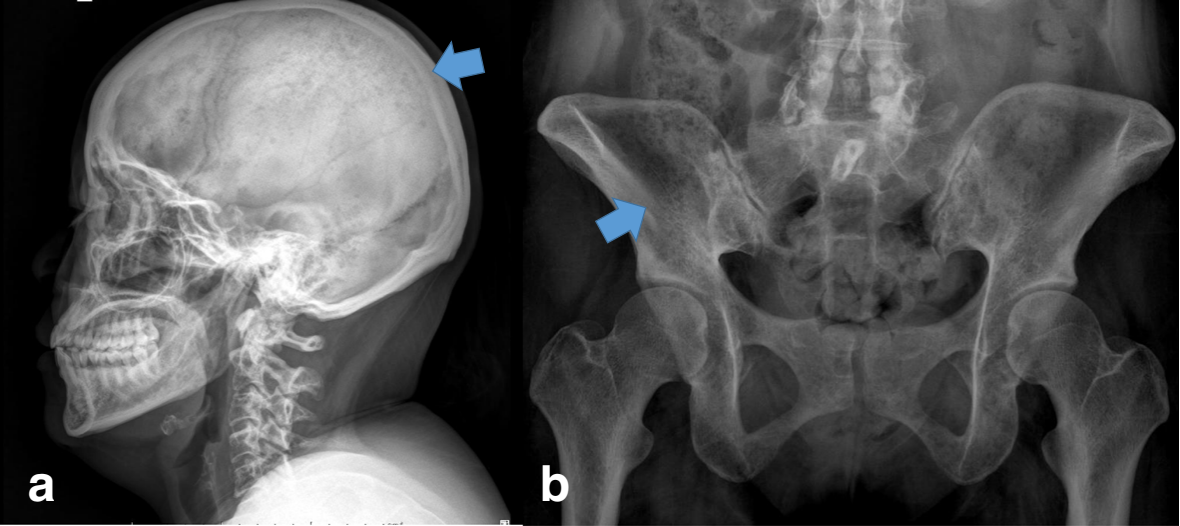
Multiple lytic bone lesions on X-rays. **a** Skull lateral X-ray (arrow). **b** Pelvis anteroposterior X-ray (arrow)

The provisional diagnosis of ECS was confirmed by a 24-hour urinary free cortisol (UFC) level of 529.4 μg/day, serum cortisol levels after 1 and 4 mg dexamethasone suppression of 26 and 25.7 mcg/dL, respectively, and a loss of physiologic diurnal variation. ACTH-independent CS was determined by an ACTH level of 3.21 pg/mL (Table 1), and computed tomography of her upper abdomen showed a lipid-poor left adrenal adenoma (size, 2.8 cm) and a lipid-poor right adrenal adenoma (size, 1.1 cm; Fig. 1). Due to the bilateral adrenal nodules, an atypical finding in adrenal CS, adrenal venous sampling (AVS) was performed to determine the potential side of the excess cortisol production. Using the reference method of William F. Young Jr *et al.*, the AVS revealed a predominantly left-sided ratio of adrenal venous to peripheral plasma cortisol (ratio, 3.21), which was compatible with a left cortisol-producing adrenal adenoma and a right, nonfunctioning adrenal adenoma [4]. In addition to the investigations for CS, plain films of the lumbosacral spine were obtained to evaluate her back pain, and they revealed a compression fracture of the T5–T10 vertebrae. Moreover, we found several osteolytic lesions in her ribs and pelvic bone that are not normally discovered in CS (Fig. 2). Metastatic cancers and MM were suspected.

**Table 1:** Tests for confirmation and subtypes of Cushing syndrome in our patient

Basal level	Results		
24-hour UFC cortisol level (mcg/day/g-Cr)	529.36		
Basal serum cortisol (mcg/dL)	30.2		
Basal plasma ACTH level (pg/mL)	3.21, 4.32, 3.43		
Diurnal variation	8:00 a.m.	4:00 p.m.	12:00 a.m.
Serum total cortisol (mcg/dL)	30.2	27.1	26.4
Dexamethasone suppression tests			
8:00 a.m. serum cortisol level after 1 mg DST (mcg/dL)	26.0		
8:00 a.m. serum cortisol level after 4 mg DST (mcg/dL)	25.7		
8:00 a.m. serum cortisol level after 8 mg DST (mcg/dL)	25.6		

*ACTH* adrenocorticotropic hormone, *DST* dexamethasone suppression test, *UFC* urinary free cortisol

**Fig. 3:**
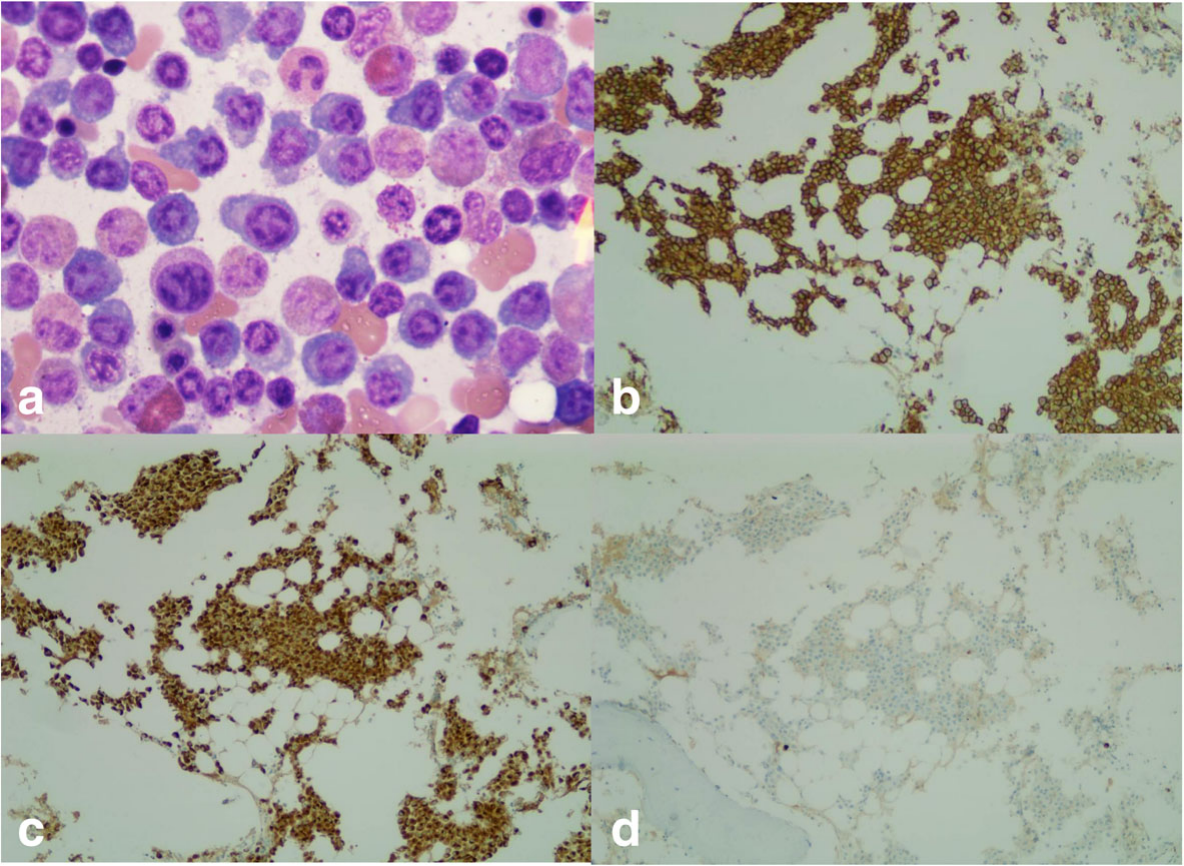
Bone marrow study at the time of diagnosis. **a** Bone marrow aspiration displaying numerous plasma cells, × 100. **b** Bone marrow biopsy showing infiltration of CD138+ plasma cells (immunostained for CD138, × 10). **c** Kappa-positive plasma cells (immunostained for kappa, × 10). **d** Lambda-negative plasma cells (immunostained for lambda, × 10)

Further diagnostic investigations were performed to identify the cause of the bone lesions and to determine if abnormal levels of ACTH were the source of the CS. A complete blood count showed hemoglobin (Hb) 10.2 g/dL, hematocrit 31.2%, white blood cells (WBCs) 10.95 × 10^9^/L with neutrophil predominating, and a normal platelet count. A peripheral blood smear displayed normochromic normocytic red cells without Rouleaux formation. There was mildly elevated serum creatinine (1.01 mg/dL), and a normal level of serum calcium (9.8 mg/dL). Serum albumin was 4.3 g/L, while serum globulin was 2.5 g/L. A bone survey revealed generalized osteolytic lesions involving her skull, mandible, both clavicles, her right scapular, both sides of her ribs, her right humerus, and her pelvic bones. A serum protein electrophoresis found hypogammaglobulinemia without detecting monoclonal gammopathy by immunofixation. The serum free kappa light chain level was 3540 mg/L, while the serum free lambda light chain level was 6.7 mg/L, with a Kappa:Lambda ratio of 531:1. A bone marrow aspiration showed plasma cells and plasmablasts over 80% and a bone marrow biopsy demonstrated numerous CD138+ plasma cells with kappa light chain restriction (Fig. 3). The serum β2-microglobulin was 2.98 mg/L. Kappa light chain MM, International Staging System stage I, was diagnosed.

**Fig. 4:**
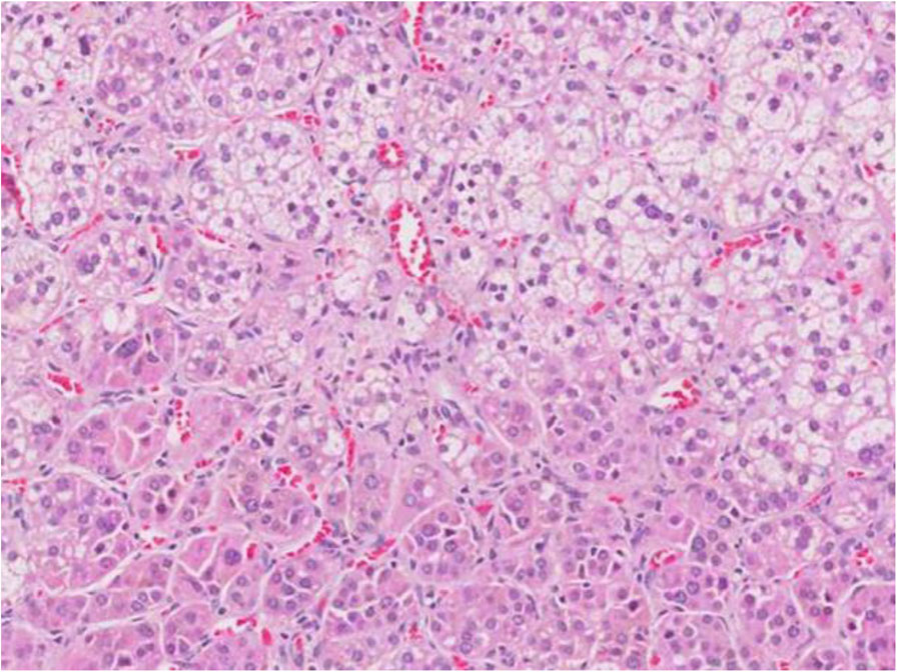
Histopathological findings from left adrenal cortical adenoma consisting of lipid-rich (clear vacuolated) cells and lipid-depleted (compact eosinophilic) cells

Because our patient had hypercortisolism, the endogenous corticosteroid might have been suppressing plasma cell proliferation in the MM. She also had no critical conditions for emergency surgical treatment of the CS. MM was therefore set as the priority treatment to eliminate the clonal plasma cells. We decided to treat her with a bortezomib, cyclophosphamide, and dexamethasone (VCD) regimen consisting of: bortezomib 2 mg intravenously on days 1, 8, 15, and 22; cyclophosphamide 400 mg orally on days 1, 8, 15, and 22; and dexamethasone 40 mg orally on days 1, 8, 15, and 22. The treatment was recycled every 28 days, with a total of six cycles. While she was receiving this chemotherapy regimen, she did not develop hyperglycemia or dyslipidemia. After six cycles, she finally achieved a very good partial response, according to the International Myeloma Working Group (IMWG) response criteria, and a stem cell collection was made after this cycle. The protocol for stem cell mobilization consisted of 4000 mg of cyclophosphamide in normal saline (250 mL) administered intravenously for 4 hours on day 1, and filgrastim 600 mcg in 5% dextrose water (100 mL) administered intravenously for 15 minutes on each of days 2–10. The stem cells were collected on days 10–11 without any negative CS effect on the stem cell collection process; the CD34+ cell count was 4.4 × 10^6^ cells/kg BW. An autologous stem cell transplant was performed in September 2014. There were no serious complications, and gained a deeper response in the form of a complete response after this intervention. Subsequently, 5 months after the stem cell transplant, a left laparoscopic adrenalectomy was successfully undertaken. The pathological report showed an adrenal cortical adenoma of size 2.0 × 2.4 × 0.6 cm (Fig. 4). Her serum cortisol level returned to 1 mcg/dL after the operation; she received a steroid replacement, which was gradually discontinued over 9 months. Her blood pressure decreased slowly, and we successfully tapered off the anti-hypertensive drugs over 2 months. Her Cushingoid appearance gradually subsided. Eventually, she was in remission of both diseases after more than 30 months of treatment. Timeline of her symptoms, treatments, and outcome was provided in Additional file 1.

## Discussion

Glucocorticoid excess, the common cause of osteoporosis, can affect the bone function and leads to a high morbidity among patients. Osteoporotic fractures and avascular necrosis are well-known skeletal manifestations of CS. This particular patient had an atypical presentation of CS that could result in misdiagnoses. Notably, she had severe bone pain in some areas that were not compatible with common sites of osteoporotic fracture, and she had multiple osteolytic lesions revealed through a bone survey. Manifestations of bone involvement in patients with MM can also present; the wide range of symptoms includes osteopenia, multiple osteolytic lesions without sclerotic rim, and fragility fractures.

A literature review revealed that only two cases of MM with CS had been reported; in our case, however, there were some marked differences in the clinical manifestations compared with those reports [5, 6]. Table 2 tabulates the previously published series of MM concomitant with CS.

**Table 2:** Reported cases of multiple myeloma coexisting with Cushing syndrome

Author and Reference number	Year	Patient	Diseases	Monoclonal gammopathy type	Treatment sequence	Treatment outcomes
Kinugawa *et al*. [5]	1989	55-year-old woman	MM with Cushing disease	IgG-lambda	- Low dose of o,p’-DDD to control hypercortisolemia - MP regimen for treatment of MM	- After cortisol level was normalized, MM symptoms were exacerbated
Kebapclar *et al*. [6]	2007	60-year-old man	MM with subclinical Cushing syndrome	IgA	- VAD regimen for MM treatment	- Not applicable
This case	2018	33-year-old woman	MM with left cortisol-producing adrenal adenoma	Kappa light chain	- VCD regimen with subsequent ASCT for treatment of MM - Left adrenalectomy	- MM in complete response - CS in remission

*CS* Cushing syndrome, *MM* multiple myeloma, *MP* melphalan/prednisolone, *o,p’-DDD* mitotane, *VAD* vincristine/doxorubicin/dexamethasone, *VCD* bortezomib/cyclophosphamide/dexamethasone, *ASCT* autologous stem cell transplantation

Kinugawa *et al.* reported a case of Cushing disease (CD) and MM [5]. The clinical features were a rounded face, central obesity, and hypertension, so CS was suspected. That patient was older than the one in our case. The confirmatory laboratory test for CS was positive with a low-dose dexamethasone suppression test, an increase in urinary excretion of 17-hydroxycorticosteroids (17-OHSC), and a loss of diurnal variation of serum cortical steroid. The 8-mg overnight dexamethasone suppression test and ACTH level were evaluated to discriminate between ACTH-dependent and ACTH-independent CS. Pituitary microadenoma was confirmed as the source of the hypercortisolism by computed tomography and inferior petrosal sinus sampling. The clinical clues to the myeloma were the bone pain and the presence of Bence−Jones proteins in the urine. In contrast, our case also had mild anemia with bone pain, which was detected as multiple osteolytic lesions in the bone survey.

Another report by Kebapclar *et al.* demonstrated subclinical CS from a right adrenal incidentaloma in a patient with myeloma [6]. The patient was a 60-year-old man who presented with a right adrenal incidentaloma (revealed by an abdominopelvic ultrasound) but without a Cushingoid appearance. Subclinical CS was confirmed by a high 24-hour UFC cortisol level (390 mcg/day), loss of diurnal variation of serum cortisol, and a low ACTH level (< 10 pg/mL). A mild anemia (Hb 10.8 g/dL) and lytic lesions (evident in skull X-rays) were clues to the MM.

Treatment consideration varied by case. With the first, the patient was treated initially for hypercortisolemia [5]. Although surgical resection of a pituitary adenoma is conventionally the first-line therapy of CD, the patient was treated with mitotane (also known as o,p’-DDD) in accordance with her preferences. Successful treatment, demonstrated by a reduction in her plasma cortisol level, led to an exacerbation of the MM, which was then treated with melphalan and prednisolone. By comparison, the sequence of our treatment was different as we preferred to manage the more aggressive disease first. We decided to initiate therapy for the MM prior to surgical resection of the cortisol-producing adrenal tumor since our patient had well-controlled blood pressure and a stable CS condition. Our patient first received a VCD regimen, and then an autologous stem cell transplantation was performed, corresponding to the standard treatment for transplant-candidate patients with MM [7, 8]. After an excellent response by the MM, our patient subsequently underwent a left adrenalectomy. She remains in a good condition. Taking all into consideration, in difficult situations such as in this case, we advocate that MM should be given priority for treatment if the CS is not presenting any serious or life-threating complications.

## Conclusion

We have reported the first case of ACTH-independent CS from a left autonomous cortisol-secreting adrenal adenoma in a patient with myeloma.

## Additional file

Additional file 1: Timeline. (DOCX 15 kb)

## Abbreviations

17-OHSC: 17-hydroxycorticosteroids
ACTH: Adrenocorticotropic hormone
AVS: Adrenal venous sampling
BW: Body weight
CD: Cushing disease
CS: Cushing syndrome
ECS: Endogenous Cushing syndrome
Hb: Hemoglobin
IMWG: International Myeloma Working Group
MM: Multiple myeloma
UFC: Urinary free cortisol
VCD: Bortezomib, cyclophosphamide, and dexamethasone
WBC: White blood cell
