# Supplementary material for: Multiple myeloma concealed by adrenal Cushing syndrome: a case report and review of the literature
Source: J Med Case Rep. 2018 Aug 13;12:218. doi: 10.1186/s13256-018-1731-y (PMC6088412; doi:10.1186/s13256-018-1731-y)
Supplement: Supplementary file 1 — Timeline. (DOCX 15 kb) [file 13256_2018_1731_MOESM1_ESM.docx]

| **Dates** | **Relevant Past Medical History and Interventions** | | |
| --- | --- | --- | --- |
| 10 February 2012 | She also had a history of occasional use of Chinese herbs and weight loss pills. Her body weight had increased by 10 kilograms over a period, and she also noticed dark striae on her abdominal wall. Furthermore, she complained of excessive acne on her face, but she still had a normal period of menstruation and no hirsutism. She did not visit any hospital before. | | |
| **Dates** | **Summaries from Initial and Follow-up Visits** | **Diagnostic Testings**  **(including dates)** | **Interventions** |
| 6 Jan 2014 | She had worsening back and right-arm pain after non-severe falling accident.  On examination, a rounded face, truncal obesity, and wide purplish striae on her abdominal wall and right thigh were observed. Her blood pressure was 160/90 mmHg. | The investigations for diagnosis of exogenous Cushing syndrome consisted of:   - Serum morning cortisol, ACTH level (6Jan2014) - 24-hour, urine free cortisol level (7Jan2014) - serum cortisol level after 1 and 4 mg dexamethasone suppression (9Jan 2014) - Computed tomography of the upper abdomen (12Jan2014) - Adrenal venous sampling (15Jan2014)   The investigations for diagnosis of multiple myeloma consisted of:   - Complete blood count and blood smear (6Jan2014) - Serum creatinine, electrolyes, calcium, serum calcium (9.8 mg/dL). Serum albumin was 4.3 g/L, while serum globulin liver function tests (6Jan2014) - Bone survey (7Jan2014) - Serum protein electrophoresis, immunofixation, serum free light chain (7Jan2014) - Bone marrow study (8Jan2014) | - Adrenal venous sampling (15Jan2014)  - Bone marrow study (8Jan2014) |
| 16 Jan 2014 | Starting chemotherapy treatment (VCD regimen) for multiple myeloma | - | VCD regimen consisted of bortezomib 2 mg intravenously on days 1, 8, 15 and 22; cyclophosphamide 400 mg p.o. on days 1, 8, 15 and 22; and dexamethasone 40 mg p.o. on days 1, 8, 15 and 22. The treatment was recycled every 28 days, with a total of 6 cycles. |
| 16 July 2014 | Multiple myeloma assessment of treatment response | - Serum protein electrophoresis, immunofixation, serum free light chain (16July2014)  - Bone marrow study (16July2014) | - Bone marrow study (16July2014) |
| 22 September 2014 | Autologous stem cell transplantation for multiple myeloma ‘s treatment | - | Autologous stem cell transplantation |
| 18 March 2015 | Treatment of adrenal cortical adenoma | Serum cortisol level after surgery (23 March 2015) | -Left laparoscopic adrenalectomy  - Steroid replacement after surgery |
| 25 May 2015 | Follow up of Cushing syndrome | - | Off anti-hypertensive drugs |
| 7 Dec 2015 | Follow up of Cushing syndrome | - | Off steroid |
| 9 Jan 2018 | Follow up of multiple myeloma and Cushing syndrome | - Serum protein electrophoresis, immunofixation, serum free light chain (9Jan2018) | No medication |
